# A Role for the Anti-Viral Host Defense Mechanism in the Phylogenetic Divergence in Baculovirus Evolution

**DOI:** 10.1371/journal.pone.0156394

**Published:** 2016-05-31

**Authors:** Toshihiro Nagamine, Yasushi Sako

**Affiliations:** RIKEN, Wako, Saitama 351–0198, Japan; Institute of Plant Physiology and Ecology, CHINA

## Abstract

Although phylogenic analysis often suggests co-evolutionary relationships between viruses and host organisms, few examples have been reported at the microevolutionary level. Here, we show a possible example in which a species-specific anti-viral response may drive phylogenic divergence in insect virus evolution. Two baculoviruses, *Autographa californica* multiple nucleopolyhedrovirus (AcMNPV) and *Bombyx mori* nucleopolyhedrovirus (BmNPV), have a high degree of DNA sequence similarity, but exhibit non-overlapping host specificity. In our study of their host-range determination, we found that BmNPV replication in *B*. *mori* cells was prevented by AcMNPV-P143 (AcP143), but not BmNPV-P143 (BmP143) or a hybrid P143 protein from a host-range expanded phenotype. This suggests that AcMNPV resistance in *B*. *mori* cells depends on AcP143 recognition and that BmNPV uses BmP143 to escapes this recognition. Based on these data, we propose an insect-baculovirus co-evolution scenario in which an ancestor of silkworms exploited an AcMNPV-resistant mechanism; AcMNPV counteracted this resistance via P143 mutations, resulting in the birth of BmNPV.

## Introduction

Baculoviruses are a family of large insect dsDNA viruses that have been described in over 600 species of holometabolous insect hosts including Diptera, Hymenoptera and Lepidoptera [1]. Each baculovirus exhibits unique host specificity and this virus classification depends frequently on its host specificities [1]. Recent phylogenomic analyses showed that baculoviruses form a monophyletic clade, in which they cluster according to their host order [2, 3]. Based on these data, a scenario regarding baculovirus evolution was proposed that ancient co-evolution between the viruses and hosts led to the progressive specialization of different baculovirus linages to hosts of different orders [3]. Thus, the long-term co-evolution between baculoviruses and insect hosts seems to contribute to the specialization of these pathogens to their hosts; however, at micro-evolutionary levels, molecular mechanisms in co-evolution are still poorly understood.

**Fig 1:**
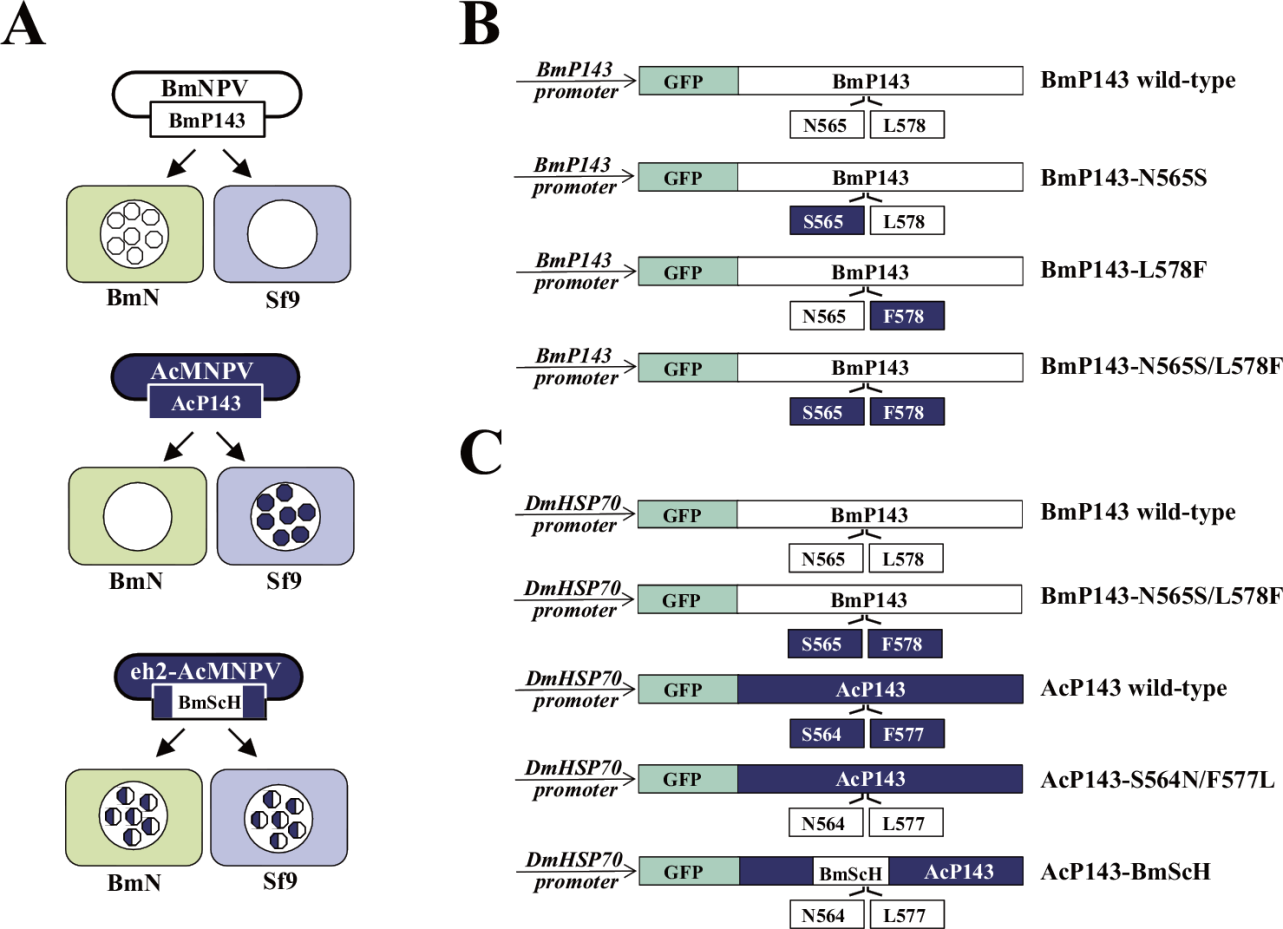
Schematic representation of viruses, cells and recombinant P143 proteins used in this study. (A) BmNPV replicates in *B*. *mori* (BmN) cells but does not in *S*. *frugiperda* (Sf9) cells, while AcMNPV replicates in *S*. *frugiperda* cells but does not in *B*. *mori* cells. The AcMNPV recombinant virus (eh2-AcMNPV), in which the host range-specificity region of AcP143 is replaced with the corresponding BmP143 sequence (BmScH), replicates in both *B*. *mori* cells and *S*. *frugiperda* cells. (B and C) The fusion proteins of GFP and P143 recombinant proteins were expressed in virally infected insect cells under the control of the authentic *BmP143* promoter (B) or under the control of the *DmHSP70* promoter (C).

The two closely related baculoviruses *Autographa californica* multiple nucleopolyhedrovirus (AcMNPV) and *Bombyx mori* nucleopolyhedrovirus (BmNPV) have a high degree of DNA sequence similarity [4], but exhibit non-overlapping host specificity [5]. AcMNPV replicates in *Spodoptera frugiperda* cells and *Trichoplusia ni* cells but does not in *B*. *mori* cells, while BmNPV replicates in *B*. *mori* cells but does not in either *S*. *frugiperda* cells or *T*. *ni* cells (reference [6] and see Fig 1A). Coinfection of *B*. *mori* cells with AcMNPV and BmNPV prevents production of both AcMNPV and BmNPV; however, coinfection of *S*. *frugiperda* cells with AcMNPV and BmNPV leads to BmNPV replication as well as AcNPV replication, generating host-range expanded viruses that are able to replicate in both types of cells [7]. Genomic analysis of these host-range expanded viruses revealed that a baculovirus DNA helicase gene, *p143*, is responsible for the host-range expansion [5, 8, 9]. AcMNPV-P143 (AcP143) and BmNPV-P143 (BmP143) share about 96% identity in their amino acid sequences [9]; however, recombinant AcMNPVs with short sequence substitutions in *Bmp143* that correspond to *Acp143* sequences exhibit the host-range expanded phenotype [5, 8, 9]. By fine mapping the host range-specificity region using silkworm larvae, it has been shown that substitution of only two amino acids, S564N and F577L, is sufficient to change the host range specificity of AcMNPV [10].

In this report, we show that BmNPV replication in *B*. *mori* cells is blocked by expression of AcP143 but not that of BmP143 or a hybrid P143 protein from a host range-expanded phenotype. Taken together with reports characterizing host range-expanded viruses [5, 8, 9], this result suggests that AcMNPV resistance in *B*. *mori* cells depends on AcP143 recognition and that BmNPV escapes this recognition by use of BmP143. Based on these data, we propose that, while an AcMNPV-resistant mechanism was exploited in an ancestor of silkworms, AcMNPV counteracted this resistance via P143 mutations, resulting in the birth of BmNPV.

## Results and Discussion

### Effect of reverse amino acid-substitutions in the P143 host-range specificity region on virus replication

**Fig 2:**
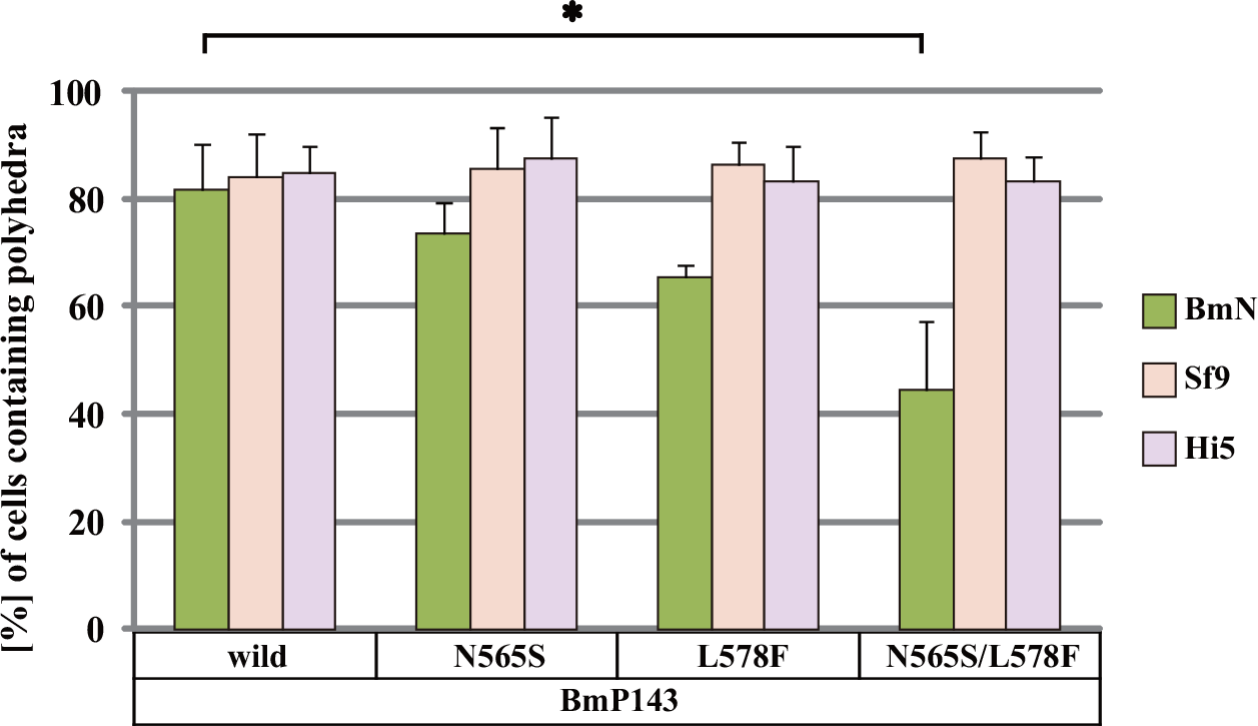
Effect of authentic *BmP143* promoter-driven BmP143 recombinant proteins on polyhedron formation. BmN, Sf9 and Hi5 cells were transfected with plasmids expressing the BmP143 recombinant proteins indicated. The transfected BmN cells were infected with wild-type BmNPV and the transfected Sf9 and Hi5 cells were infected with wild-type AcMNPV. At 72 hpi, the proportion of polyhedron-containing cells was calculated under a fluorescence microscope. Average of three experiments ± SEM are shown. Student's *t*-test: (*) *p* < 0.05. ANOVA: BmN *p* = 0.0098; Sf9 *p* = 0.959; Hi5 *p* = 0.893.

Recombinant AcMNPVs with short sequence substitutions in the *p143* locus corresponding to the *Bm-p143* (572 bp or less) sequence exhibit the host range-expanded phenotype [5, 8, 9]. In addition to cultured cell experiments, *in vivo* studies using silkworm larvae revealed that substitution of only two amino acids, S564N and F577L, within the P143 host-range specificity region is sufficient to change the specificity, i. e., the recombinant AcMNPV become infectious to *B*. *mori* larvae [10]. To explore a role for the two amino acid residues Ser564 and Phe577 in host restriction, we constructed plasmids that express fusion proteins of GFP and BmP143 mutants bearing the reverse substitution of the expanded host range phenotype (N565S/L578F, N565S, and L578F) under the control of the authentic *Bm-p143* promoter (Fig 1B) as we had previously demonstrated that GFP has no effect on the BmP143 function [11]. When *B*. *mori* (BmN) cells were transfected with the single amino acid mutant, N565S or L578F, and then infected with wild type BmNPV, a slight suppression was observed; however, there was no significant difference between wild type BmP143 protein and each mutant protein in polyhedron formation efficiency (N565S, *p* = 0.318; L578F, *p* = 0.057) (Fig 2). In contrast, cells transfected with the double mutant N565S/L578F had a significant reduction in efficiency (*p* = 0.0255). This result suggests that Ser565 and Phe578 in the BmP143 mutant (and possibly Ser564 and Phe577 in AcP143) have a synergistic inhibitory effect in BmN cells.

On the other hand, none of the four proteins, wild type BmP143, N565S, L578F or N565S/L578F, had significant effects on AcMNPV replication in *S*. *frugiperda* (Sf9) cells or *T*. *ni* (Hi5) cells (Fig 2). This result is consistent with the fact that BmNPV has no inhibitory effect on AcMNPV replication in these cells [6]. Taken together with the fact that recombinant AcMNPV with S564N and F577L mutations in P143 is infectious to *B*. *mori* larvae, it is possible that Ser564 and Phe577 in AcP143 are important factors for the AcMNPV-induced inhibition of BmNPV replication in *B*. *mori* cells.

### Lack of an AcP143 recombinant protein-mediated inhibitory effect on the expanded host range phenotype of BmNPV replication in *B*. *mori* cells

**Fig 3:**
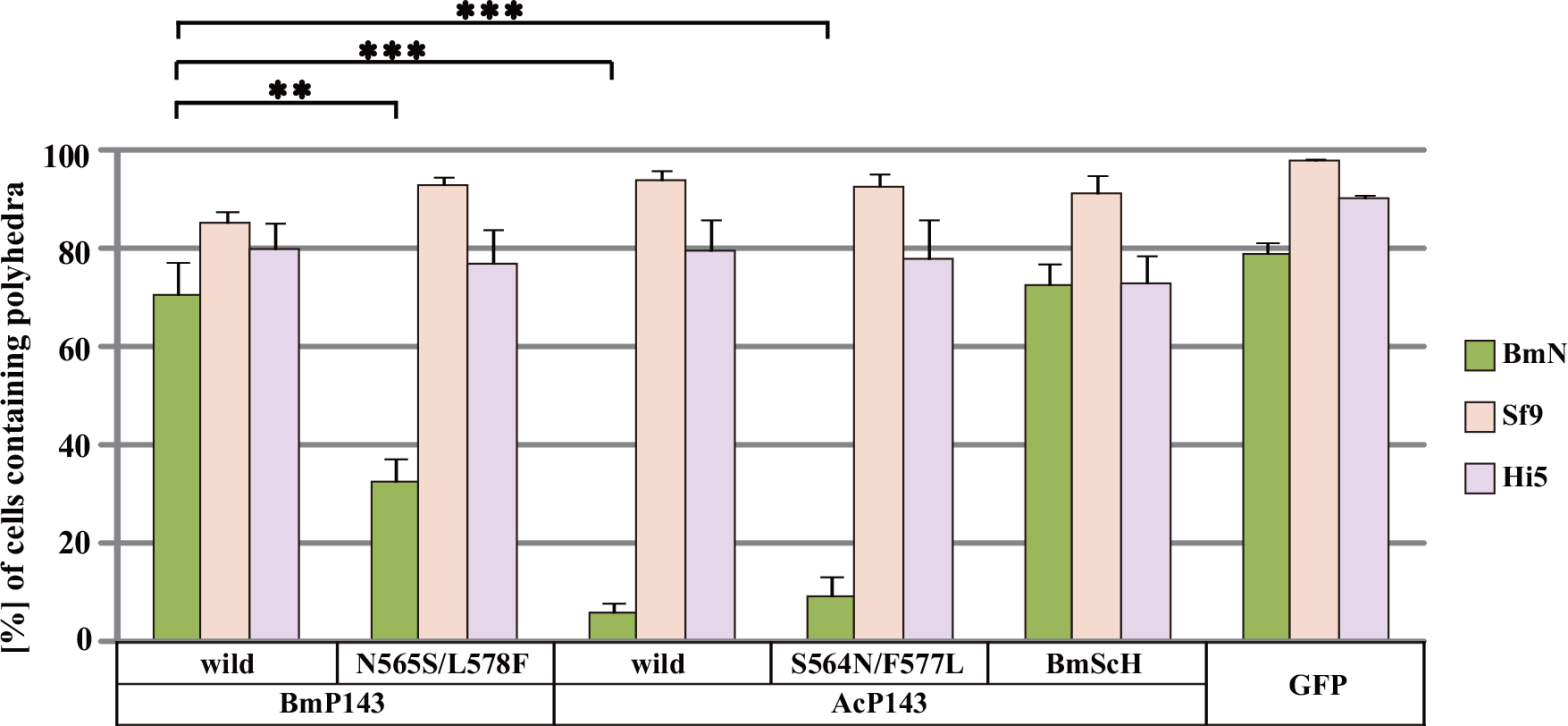
Effect of *DmHSP70* promoter-driven P143 recombinant proteins on polyhedron formation. BmN, Sf9 and Hi5 cells were transfected with plasmids expressing the P143 recombinant proteins indicated. The transfected BmN cells were infected with wild-type BmNPV and the transfected Sf9 and Hi5 cells were infected with wild-type AcMNPV. At 72 hpi, the proportion of polyhedron-containing cells was calculated under a fluorescence microscope. Average of three experiments ± SEM are shown. Student's *t*-test: (**) *p* < 0.01; (***) *p* < 0.001. ANOVA: BmN *p* = 4.2 x 10^−8^; Sf9 *p* = 0.0453; Hi5 *p* = 0.828.

Despite killing *B*. *mori* larvae, recombinant AcMNPV with the S564N and F577L mutations in P143 does not replicate in cultured *B*. *mori* cells [10]. To examine whether inhibitory factors other than Ser564 and Phe577 are present in AcP143, especially in the assay using cultured cells, we constructed an additional five plasmids expressing various types of P143 proteins (Fig 1C). In this experiment, the *Drosophila melanogaster* heat shock protein 70 (*HSP70*) promoter was used because a BmNPV-originated promoter may function more effectively in *B*. *mori* cells than in *S*. *frugiperda* or *T*. *ni* cells. Similar to the above experiment (Fig 2), expression of BmP143-N565S/L578F suppressed polyhedron formation in BmNPV-infected BmN cells, but not in AcMNPV-infected Sf9 cells or Hi5 cells (Fig 3). A slight difference between the two experiments (i. e., the authentic *BmP143* promoter-driven expression [Fig 2] and *DmHSP70-*promoter-driven expression [Fig 3]) was observed in polyhedron formation efficiency of BmP143-N565S/L578F-expressing cells (45% vs. 33%) as well as wild-type BmP143-expressing cells (82% vs. 71%). This may be due to earlier expression from the *DmHSP70* promoter than from the *BmP143* promoter, which functions as a viral delayed early gene while the *DmHSP70* promoter initiates expression prior to viral infection under our experimental condition. Unlike the moderate effects of BmP143-N565S/L578F, wild-type AcP143 blocked polyhedron formation almost completely. Moreover, while the S564N/F577L double mutation in AcP143 makes AcMNPV infectious to *B*. *mori* larvae [10], AcP143-S564N/F577L blocked BmNPV replication in cultured *B*. *mori* cells. These results suggest that amino acid-residues other than Ser564 and Phe577 in AcP143 have an inhibitory effect on BmNPV replication in cultured BmN cells rather than in *B*. *mori* larvae. We therefore examined a P143 recombinant protein from a virus that exhibits the expanded host range phenotype in both *B*. *mori* larvae and cultured cells [5]. This protein consists of the full length AcP143 protein except for the ScH region (413–602 aa), which was replaced with BmP143-ScH (414–603 aa) [5] (Fig 1C and S1 Fig). In contrast to AcP143 or AcP143-S564N/F577L, expression of this hybrid P143 protein had no inhibitory effect on polyhedron formation in BmNPV-infected BmN cells (Fig 3). This result suggests that replication-inhibiting factors in cultured cells are confined within the AcP143-ScH region in which 14 amino acid residues, including Ser564 and Phe577, differ from the BmP143-ScH sequence [5, 9] (S1 Fig). While BmP143-N565S/L578F has a significant effect on BmNPV replication, AcP143-S564N/F577L impedes viral replication (Fig 3), which implies that the AcP143-ScH region has redundant toxicity on BmNPV replication, that is, the multifaceted 3D-conformation of this region could affect BmNPV-infected BmN cells redundantly. This redundancy, however, may be specific in cultured cells because the S564N/F577L mutation in AcP143 leads to AcMNPV replication in *B*. *mori* larvae, i.e., AcP143-S564N/F577L is likely non-toxic in this insect larvae [10]. Similar to the apparent discrepancy in infectivity of the recombinant virus (AcMNPV-AcP143-S564N/F577L) between *B*. *mori* larvae and cultured cells, it remains unclear whether the differences in toxicity in *B*. *mori* larvae and cultured cells for this mutated protein are due to differences in tissue susceptibility or simply greater permissivity of *B*. *mori* larvae [10]. Possibly, both of the two reasons might be involved in the apparent discrepancy, i.e., several types of cells could be more permissive to AcP143-S564N/F577L within *B*. *mori* larvae than in vitro.

### An evolutionary model for the phylogenetic separation between AcMNPV and BmNPV

**Fig 4:**
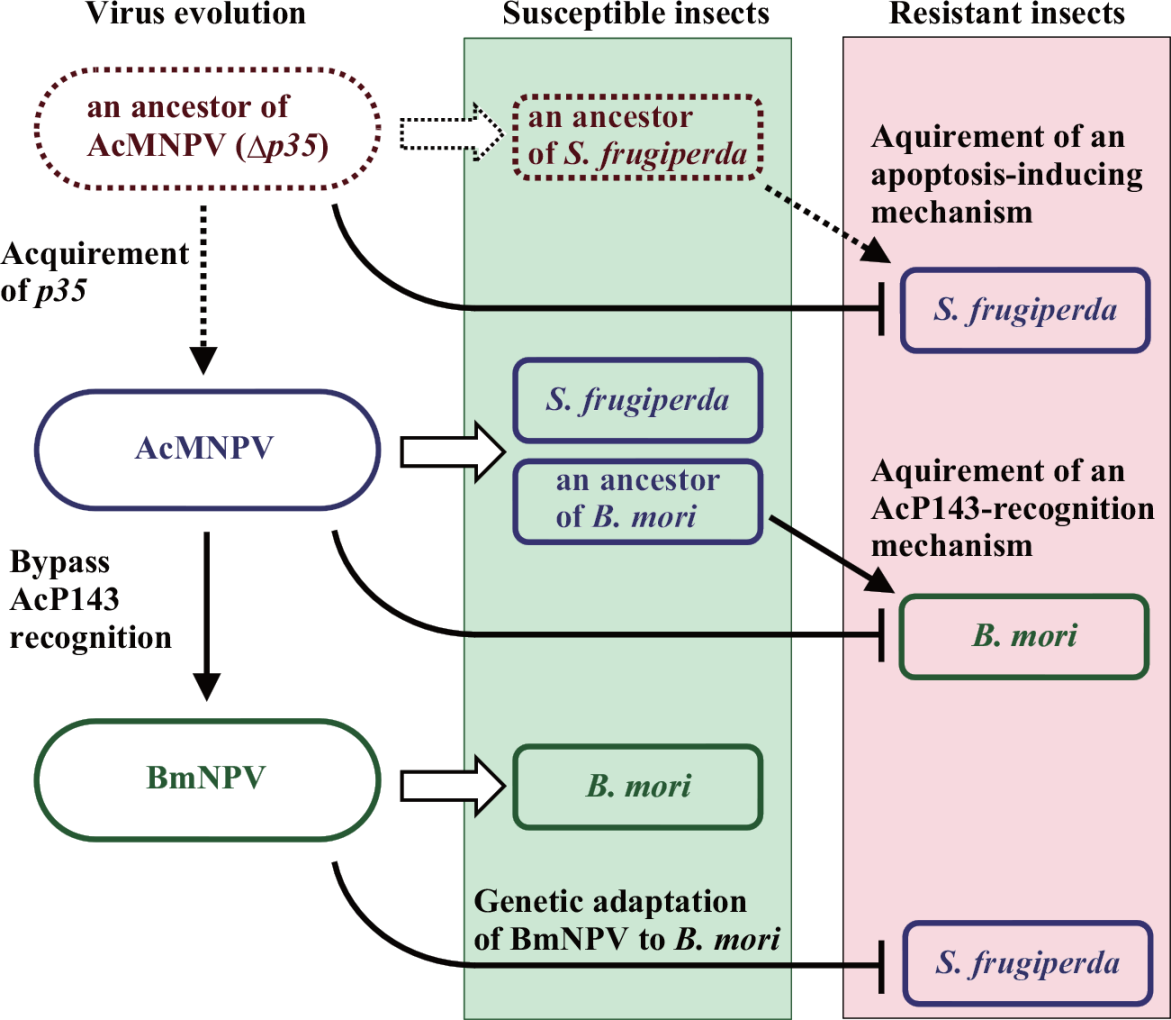
A possible scenario illustrating phylogenetic separation of BmNPV from AcMNPV. See text for details.

Since *B*. *mori* is believed to be a domesticated animal, BmNPV must have separated from AcMNPV or a closely related virus relatively recently, i.e., thousands of years ago. However, the two viruses have quite different host range specificities; AcMNPV displays a relatively broad host spectrum, whereas BmNPV’s spectrum is extremely narrow [6]. It has been unknown why such a specific change to host range spectrum occurs with BmNPV despite high similarity of the two virus genomes (> 90%) [4]. Based on the AcP143-induced blockage of BmNPV (and AcMNPV) replication, however, it might be possible to explain the specific evolution because this response in *B*. *mori* cells could function as an AcMNPV-resistant mechanism (Fig 4). Once an ancient *B*. *mori* exploited the virus defense mechanism, the number of these AcMNPV-resistant *B*. *mori* individuals likely began to predominate either naturally or artificially. In this situation, some local groups of AcMNPV might have evolved a counteraction to the AcP143-induced defense mechanism, even though their growth rates were reduced in other insects as well as conventional (i.e., AcMNPV-susceptible) *B*. *mori*. Consequently, an ancient AcMNPV (and ancestor of BmNPV) selected a mutated ScH region to avoid the AcP143-recognition mechanism in *B*. *mori* cells that concomitantly reduced viral growth rate in Sf9 cells [12]. After overcoming the AcP143-induced defense mechanism, the ancestral BmNPV continued to co-evolve with *B*. *mori* to adapt to its unique physiological and ecological conditions that rapidly changed due to heavy influence from artificial selection. As a consequence, the present BmNPV is unable to infect insects other than *B*. *mori* or its close relatives (Fig 4). *Bombyx mandarina*, a close relative of *B*. *mori*, is susceptible to BmNPV but resistant to AcMNPV [13]. Nevertheless, a wild strain of *B*. *mandarina* NPV isolated from the insect is able to infect *S*. *frugiperda* and *T*. *ni* cells as well as *B*. *mori* cells [14]. Despite possession of the P143-ScH region identical to BmP143 (S1 Fig), this virus may retain character similar to an ancient BmNPV that was inexperienced in adaptation to *B*. *mori*. While our study did not conduct the identification of toxic amino acids in AcP143-ScH region due to its redundant effects on BmNPV replication, a recent report suggests that the AcP143-ScH region is involved in the degradation of ribosomal RNA in *B*. *mori* cells [15].The degradation mechanism might contribute to the AcMNPV resistance in *B*. *mori* cells. AcP143 seems to have a suppressive effect on the expression of late genes in BmNPV-infected *B*. *mori* cells (S2 Fig).

This type of viral evolution (i. e., host defense-overcoming evolution) might also occur in the generation of AcMNPV. Deletion of the anti-apoptotic *p35* gene, one of the characteristic genes in the AcMNPV genome, reduces infectivity and induces apoptosis in AcMNPV-susceptible insects such as *S*. *frugiperda* [16]. Based on this fact, one could assume the following evolutionary scenario (Fig 4): prior to acquisition of *p35*, an ancestor of AcMNPV was capable of infecting an ancestor of *S*. *frugiperda* or related insect(s) that lacked the AcMNPV-induced apoptosis mechanism. In turn, this *S*. *frugiperda* became resistant to the ancestral AcMNPV by selecting for the apoptotic mechanism. AcMNPV then countered this defense with *p35*. Although this scenario may be too simple, it is likely that AcMNPV evolution was involved in the host apoptotic response and P35. Notably, BmNPV mutants lacking the *Bm-p35* gene induce apoptosis in *S*. *frugiperda* cells and *T*. *ni* cells rather than *B*. *mori* cells [17] (S3 Fig). It could be assumed that the loss of the BmNPV- (and AcMNPV-) induced apoptosis mechanism in *B*. *mori* cells was due to acquirement of the novel AcP143-induced defense mechanism that might hijack a part of the signaling pathway in the conventional virus-induced apoptosis mechanism.

Having genotoxic effects on host cells, nuclear resident viruses may need to frequently circumvent host apoptotic mechanisms. Studies on baculovirus evolution can provide a fruitful model for understanding host-and-virus co-evolution in regard to pro- and anti-apoptotic strategies.

## Summary

Since each baculovirus has a unique host range spectrum, studies on their host range determination mechanisms are important for not only baculovirus application such as biological pest control but also basic baculovirus research such as host-virus co-evolution. In this view, we focused on the host range determination mechanisms of AcMNPV and BmNPV as two viruses that have non-overlapping host spectrums despite similar genomes. In this report, we found that the host-range specific region in AcP143 has a toxic effect on *B*. *mori* cells but not *S*. *frugiperda* cells or *T*. *ni* cells and consequently concluded that this region is a target for a novel virus defense in *B*. *mori*, i. e., a recognition site for the *B*. *mori*-specific AcMNPV-resistant mechanism. Based on these data, in addition to previous reports, we propose the following evolutionary scenario: since *B*. *mori* acquired the AcMNPV-resistant mechanism, one group of AcMNPV, which became an ancestor of BmNPV, countered this mechanism via mutation of the recognition site. Furthermore, because BmNPV mutants lacking the *Bm-p35* gene induce apoptosis in *S*. *frugiperda* cells and *T*. *ni* cells but not in *B*. *mori* cells, we speculate that the novel AcMNPV-resistant mechanism hijacked a part of the signaling pathway in the conventional AcMNPV-induced apoptosis mechanism.

## Materials and Methods

### Cells and viruses

BmN cells were maintained in TC100 medium (Funakoshi Co., Tokyo, Japan) supplemented with 10% FBS [18]. Sf9 cells and High Five cells (Invitrogen) were maintained in SF900-II (Invitrogen) [19]. The BmNPV wild-type isolate T3 [6] and BmNPV *p35*-deleting mutants [17] were propagated in BmN cells. AcMNPV wild-type isolate E2 [20] and AcMNPV *p35*-deleting mutants [16] were propagated in Sf9 cells and High Five cells respectively.

### Plasmid construction

*BmP143* promoter-driven expression plasmids that carry recombinant genes for GFP-BmP143^N565S^, GFP-BmP143^L578F^, and GFP-BmP143^N565S/L578F^ (pPEX-GFP-P143NS, pPEX-GFP-P143LF and pPEX-GFP-P143NSLF, respectively) were constructed by site-directed mutagenesis from pPEX-GFP-P143 [11]. To construct the plasmids for *DmHSP70* promoter-driven expression of GFP-BmP143 and GFP-BmP143^N565S/L578F^ (pKm-GFP-BmP143 and pKm-GFP-BmP143-N565S/L578F, respectively), P143-ORF sequences were amplified from pPEX-GFP-P143 and pPEX-GFP-P143NSLF by PCR with primers (5'-GGAGATCTTTAACATACAAAATTTGGTA-3' and 5'-GGCTCGAGATGATTGACAACATTTTACA-3') and KOD (+) DNA polymerase (Toyobo) under the following conditions: 94°C for 2min and 35 cycles of 98°C for 10 s, 55°C for 30 s and 68°C for 6 min. The resulting PCR products were digested with BglII and XhoI and inserted into the SalI-BamHI sites of pHE-C [21], a plasmid derived from pEGFP-C1 (Clontech). The plasmids for *DmHSP70* promoter-driven expression of GFP-AcP143, GFP-AcP143^S564N/F577L^ and GFP-AcP143^BmScH^ (pKm-GFP-AcP143, pKm-GFP-AcP143-S564N/F577L, and pKm-GFP-ehP143, respectively) were constructed in the same manner as pKm-GFP-BmP143 except for PCR templates; pPIGA3hr5/Ac-p143 [22] for pKm-GFP-AcP143, pPIGA3hr5/Ac-p143-S564N/F577L for pKm-GFP-AcP143-S564N/F577L, and genomic DNA of eh2-AcNPV [7] for pKm-GFP-ehP143. pPIGA3hr5/Ac-p143 and pPIGA3hr5/Ac-p143-S564N/F577L were provided by Motoko Ikeda (Nagaya University, Japan).

### Measurement of polyhedron formation efficiency

Cells (0.5 ~ 1 x 10^6^) were seeded onto 27-mm glass-bottom dishes (Matsunami, Tokyo, Japan) and allowed to stand for several hours for cell attachment. To introduce plasmids, the cells were transfected with 0.5 μg of each plasmid DNA sample by using Lipofectin reagent (Invitrogen). The transfected cells were incubated at 28°C for 24 hours and infected with virus at a multiplicity of infection (MOI) of 10. At 72 hpi, the proportion of polyhedron-containing cells / GFP-expressing cells was calculated under a fluorescence microscope.

## Supporting Information

S1 Fig Amino acid sequence alignment of the P143-ScH regions from five nucleopolyhedroviruses; *Bombyx mori* NPV (*B*. *mori*), *Bombyx mandarina* NPV (*B*. *mandarina*), *Autographa californica* MNPV (*A*. *californica*), *Maruca vitrata* NPV (*M*. *vitrata*) and *Antheraea pernyi* NPV (*A*. *pernyi*). Sequences that differ from the *Bombyx mori* NPV P143 sequence are indicated for each amino acid residue, whereas identical sequences are represented by dots. The amino acid residues targeted for mutagenesis in this experiment are marked by asterisks.
(PDF)

S2 Fig AcMNPV-P143 (AcP143) reduces late-gene expression and polyhedron formation in BmNPV-infected *B*. *mori* cells. BmN cells were transfected with a plasmid expressing GFP-BmP143 (BmP143) or GFP-AcP143 (AcP143) in conjunction with a plasmid expressing Halo-tagged BmNPV-P74 under the control of the authentic *p74* promoter before infection with a wild-type of BmNPV. After 24 (24 hpi) and 48 h postinfection (48 hpi), the infected cells were stained with Halo Tag TMR Ligand (Promega) and analyzed by confocal microscopy.
(PDF)

S3 Fig *B*. *mori* (BmN), *S*. *frugiperda* (Sf9) and *T*. *ni* (Hi5) cells infected with BmNPV- and AcMNPV-mutants lacking the anti-apoptotic gene *p35*. (PDF)
