# Supplementary material for: A Role for the Anti-Viral Host Defense Mechanism in the Phylogenetic Divergence in Baculovirus Evolution
Source: PLoS One. 2016 May 31;11(5):e0156394. doi: 10.1371/journal.pone.0156394 (PMC4887030; doi:10.1371/journal.pone.0156394)
Supplement: S1 Fig — Sequences that differ from the Bombyx mori NPV P143 sequence are indicated for each amino acid residue, whereas identical sequences are represented by dots. The amino acid residues targeted for mutagenesis in this experiment are marked by asterisks. (PDF) [file pone.0156394.s001.pdf]

Supplementary Information

A ROLE FOR THE ANTI-VIRAL HOST DEFENSE MECHANISM IN THE PHYLOGENETIC DIVERGENCE IN BACULOVIRUS EVOLUTION

Authors: Toshihiro Nagamine, Yasushi Sako

```
B.mori         414  ALKRCEPYKKQEKVVFNRTCNHWFDFDDTKLCVSLGYYYGIHYMIYLTLSAKNETLDDDELWAYT  478
B.mandarina    414  ·················································································  478
A.californica  413  ·············I····A···················F·········Q·····I··H·······  477
M.vitrata      411  ···········DRI····A·······E·N··S······F···F·····Q······S·F·······  475
A. pernyi      407  ··E··R·FTTRGVAI··NL·D··HC·KGVNPY·M··S···A··F···KF·SSDAH-EC·DP··F·  470

B.mori         479  YENVMALNLPPDIVCKGFFRKLENVVTGVNLVFNGKNYQIVKKEDDLFKLVKSNCYKLSNIKFNN  543
B.mandarina    479  ·················································································  543
A.californica  478  ····································H·············T··············  542
M.vitrata      476  ·······K·SS·························H·············T····R········  540
A. pernyi      471  ·K·A·ECEV·LLVLGQA··I·V····Q·T·I···EH······D···Y··FNN·P···Q······  535

                                         *            *
B.mori         544  WKYLYLTTYGVYNLFTNSFHSNCPFLLGTTLPQTLKKPTDEEYLPEDAFNYMLSTSTDEL       603
B.mandarina    544  ············································································       603
A.californica  543  ········H····V·······S············F······K··············A···       602
M.vitrata      541  ········H····V·······S············F······K··SD··········V···       600
A. pernyi      536  ···M·H·K·····VI·DE·Y··········M·G·F·R·D·PP·····V·A···T··AE·R       595
```

S1 Fig. Amino acid sequence alignment of the P143-ScH regions from five nucleopolyhedroviruses; *Bombyx mori* NPV (*B. mori*), *Bombyx mandarina* NPV (*B. mandarina*), *Autographa californica* MNPV (*A. californica*), *Maruca vitrata* NPV (*M. vitrata*) and *Antheraea pernyi* NPV (*A. pernyi*). Sequences that differ from the *Bombyx mori* NPV P143 sequence are indicated for each amino acid residue, whereas identical sequences are represented by dots. The amino acid residues targeted for mutagenesis in this experiment are marked by asterisks.
