# Supplementary material for: A Role for the Anti-Viral Host Defense Mechanism in the Phylogenetic Divergence in Baculovirus Evolution
Source: PLoS One. 2016 May 31;11(5):e0156394. doi: 10.1371/journal.pone.0156394 (PMC4887030; doi:10.1371/journal.pone.0156394)
Supplement: S2 Fig — BmN cells were transfected with a plasmid expressing GFP-BmP143 (BmP143) or GFP-AcP143 (AcP143) in conjunction with a plasmid expressing Halo-tagged BmNPV-P74 under the control of the authentic p74 promoter before infection with a wild-type of BmNPV. After 24 (24 hpi) and 48 h postinfection (48 hpi), the infected cells were stained with Halo Tag TMR Ligand (Promega) and analyzed by confocal microscopy. (PDF) [file pone.0156394.s002.pdf]

Supplementary Information

A ROLE FOR THE ANTI-VIRAL HOST DEFENSE MECHANISM IN THE PHYLOGENETIC DIVERGENCE IN BACULOVIRUS EVOLUTION

Authors: Toshihiro Nagamine, Yasushi Sako

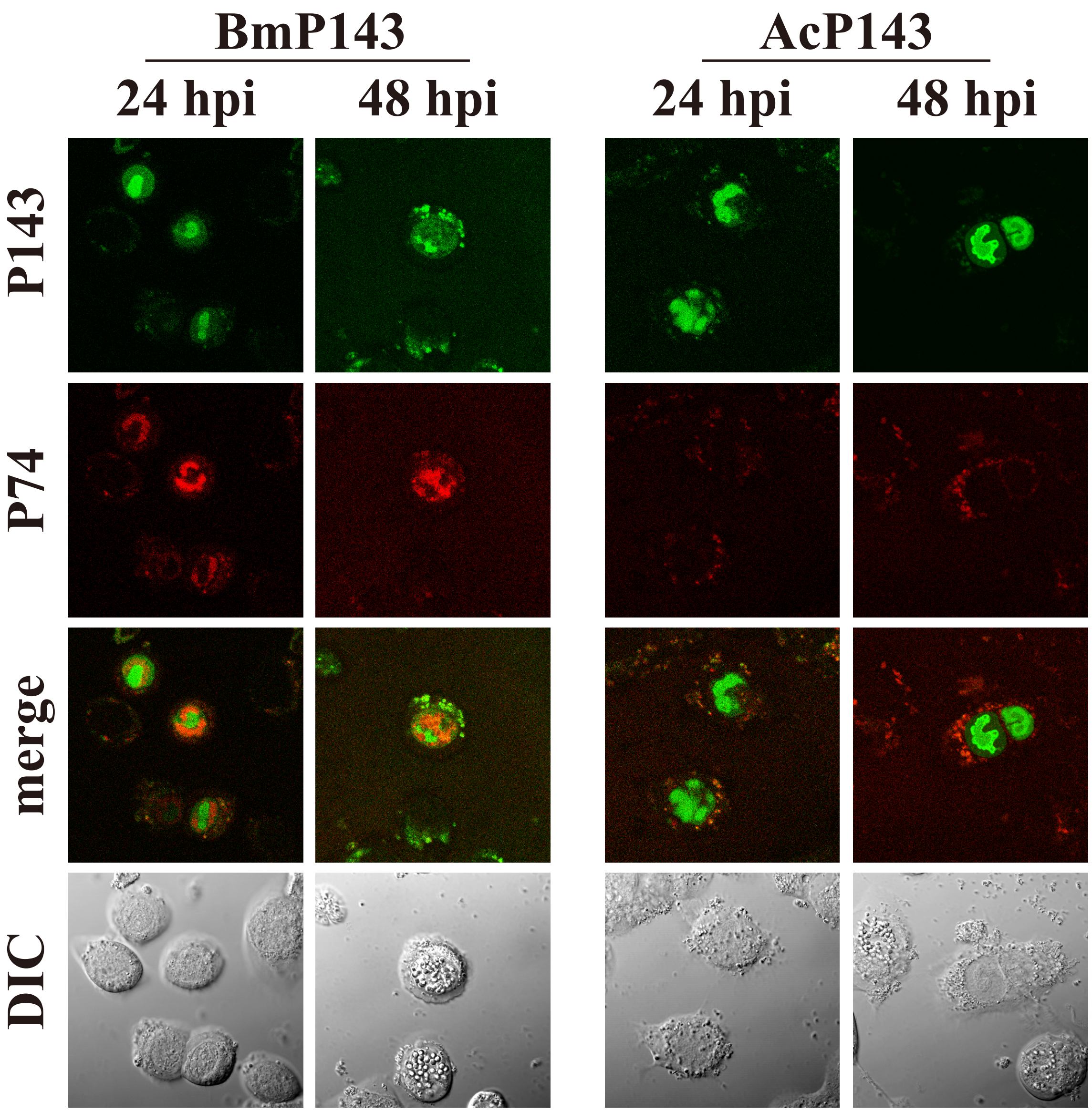

S2 Fig. AcMNPV-P143 (AcP143) reduces late-gene expression and polyhedron formation in BmNPV-infected *B. mori* cells. BmN cells were transfected with a plasmid expressing GFP-BmP143 (BmP143) or GFP-AcP143 (AcP143) in conjunction with a plasmid expressing Halo-tagged BmNPV-P74 under the control of the authentic *p74* promoter before infection with a wild-type of BmNPV. After 24 (24 hpi) and 48 h postinfection (48 hpi), the infected cells were stained with Halo Tag TMR Ligand (Promega) and analyzed by confocal microscopy.
