# Supplementary material for: A Role for the Anti-Viral Host Defense Mechanism in the Phylogenetic Divergence in Baculovirus Evolution
Source: PLoS One. 2016 May 31;11(5):e0156394. doi: 10.1371/journal.pone.0156394 (PMC4887030; doi:10.1371/journal.pone.0156394)
Supplement: S3 Fig — (PDF) [file pone.0156394.s003.pdf]

Supplementary Information

A ROLE FOR THE ANTI-VIRAL HOST DEFENSE MECHANISM IN THE PHYLOGENETIC DIVERGENCE IN BACULOVIRUS EVOLUTION

Authors: Toshihiro Nagamine, Yasushi Sako

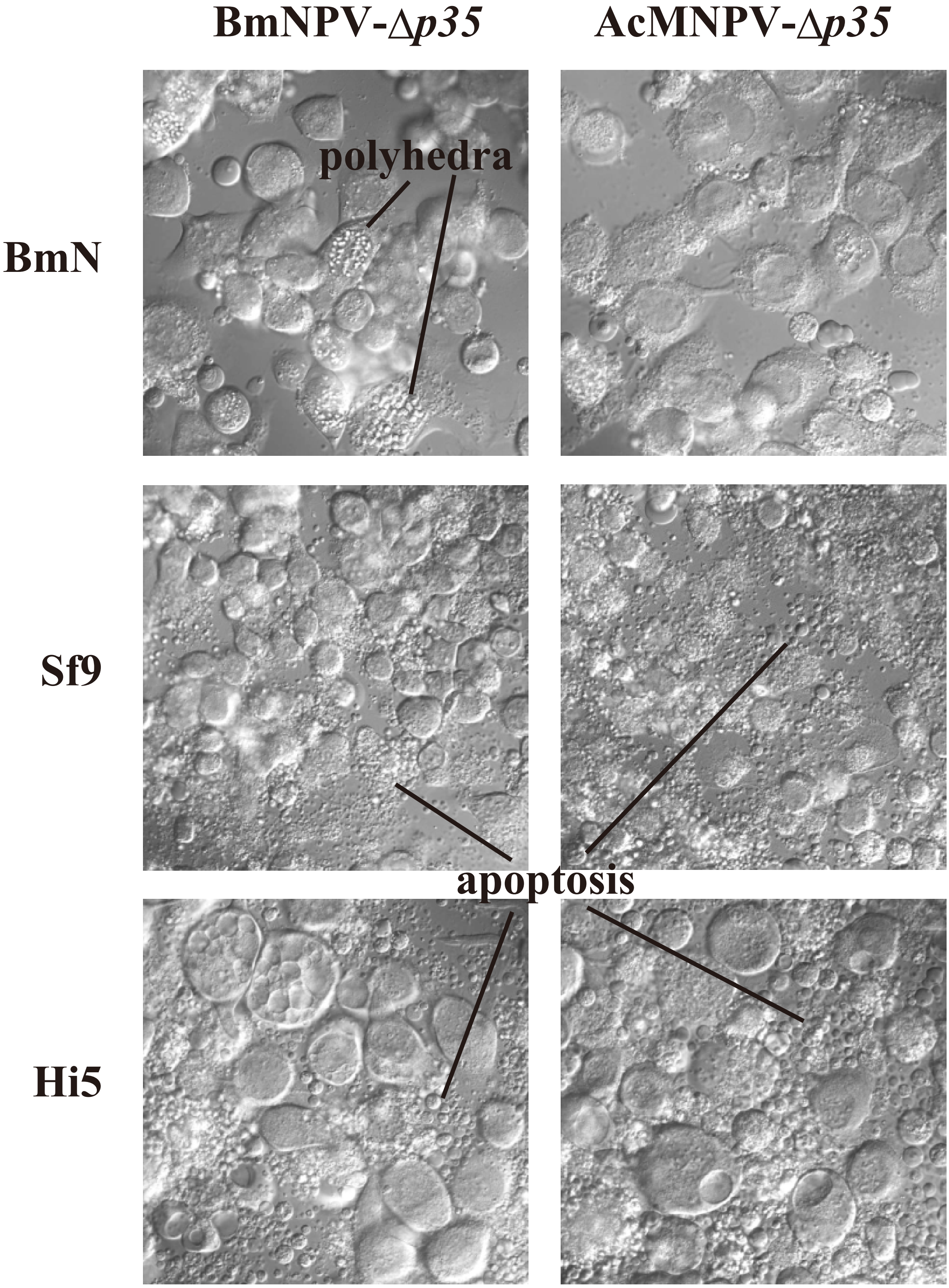

S3 Fig. *B. mori* (BmN), *S. frugiperda* (Sf9) and *T. ni* (Hi5) cells infected with BmNPV- and AcMNPV-mutants lacking the anti-apoptotic gene *p35*.
